# Meaning in life as a predictor of the general health among medical sciences students: A cross‐sectional study

**DOI:** 10.1002/nop2.731

**Published:** 2020-12-04

**Authors:** Zohreh Shahhosseini, Zeinab Hamzehgardeshi, Rahmatollah Marzband, Marzieh Azizi

**Affiliations:** ^1^ Sexual and Reproductive Health Research Center Mazandaran University of Medical Sciences Sari Iran; ^2^ Department of Islamic Studies Mazandaran University of Medical Sciences Sari Iran; ^3^ Student of Reproductive Health School of Nursing and Midwifery Tehran University of Medical Sciences Tehran Iran

**Keywords:** general health, meaning in life, predictors, students

## Abstract

**Aim:**

To investigate the predictive role of meaning in life on the general health among medical sciences students.

**Design:**

A cross‐sectional study.

**Methods:**

Four‐hundred medical sciences students were selected by proportional to size sampling. The general health questionnaire and the personal meaning profile were administrated to identify the predictive role of meaning in life on the student's general health. Descriptive statistics and linear regression were administrated.

**Results:**

The mean ± *SD* of the total score of general health and meaning in life among students was 16.34 ± 6.73 and 71.50 ± 9.78, respectively. In the multiple linear regression model, factors such as father's educational level, and meaning in life can predict a 20% variance of general health among university students. As meaning in life thus emerges as a variable worth further in the health of university students, implementing interventional studies to assess the effect of meaning in life on students' general health is recommended.

## 1 INTRODUCTION

According to the WHO definition, general health (GH) as a substantial basis of life, consisted of appropriate physical, mental and social health, not the absence of disease or disability. Over the recent decades, GH assessments are considered an important issue in educational settings (Pekmezovic et al., 2011) and its evaluation is necessary to identify the main health problems in the population (Hashemian et al., 2020).

Health‐related sciences are significant issues in the promotion of the health and welfare in the societies. The health is a complex term and consists of many types (Moghadam et al., 2014). Literature revealed that three types of possible definition for health are used in the studies (World Health Organization, 2004). According to the first definition, it is the absence of any disease or impairment. The second definition is a state that allows the individual to adequately cope with all demands of daily life, and based on third definition, health is a state of the balanced interaction that individual creates between himself and physical and social environment (Sartorius, 2006).

Student life is a critical and important period in life cycle of young and active population of any country and is often associated with significant social and personal changes in their lives (Soltani, 2016). Also, academic education may be a stressful period for university students, and in this time, they may expose to various environmental and psychological stressors as a result of concern about achievement to their academic goals, increased academic workload, peer's competitive environment and undesirable social relationships (El Ansari et al., 2013; Hussain et al., 2013; Zaid et al., 2007). Hence, pay attention to the mental health needs of the students as a considerable population group of each country is an important matter. Since, students' health status significantly predicted their academic achievements and the sustainable development of a community, identifying the associated factors affecting university students' GH is so important (Jackson & Cole, 2013).

Studies showed that medical sciences students, compared to the other university students, have more challenges such as mental and emotional stress about the hospital environment, dealing with patients' issues and diseases, the longer period of education (in the case of medical students) and concern of specific professional careers in the future. So they may be at higher risk of GH problems (Khazir & Abbasi‐Shavazi, 2019).

Meaning in life (MIL) is one of the most complex issues which occupies the minds of many theorists. Despite numerous definitions in the field of MIL by philosophers and often anthropologist and existentialist psychologists; however, there is no universal comprehensive accepted definition (Doğan et al., 2012; Wong, 1998). In this way, it is stated that MIL is a complex set of values, beliefs, feelings and conceptual schemas in which individual feels their life purposeful and significance (Mohamad et al., 2011; Steger et al., 2009) and striving towards reaching to the determined goals (Rathi & Rastogi, 2007). A meaningful life is associated with overcoming adverse situations, personal satisfaction and self‐sufficiency, psychological well‐being, happiness, self‐confidence, self‐esteem and positive affect (Kim et al., 2005; Steger et al., 2006, 2009; Steger & Kashdan, 2007). Realization of these characteristics in the students, need to draw a comprehensive image of the world, search for a meaningful life and identify the potential sources of MIL in their own life (Brassai et al., 2012). In return, meaningless life leads to the individual loss his ability to recognize himself as an important or useful factor and this issue probably associated with some psychiatric disorders such as depression, anxiety, feelings of guilt, aggression, frustration, psychological distress and existential emptiness (Doğan et al., 2012; Zika & Chamberlain, 1992).

## 2 BACKGROUND

Studies showed that there is a significantly positive relationship between mental and physical health (El Ansari et al., 2013; Savadpour & Nasiri, 2015). Mental health concept as an important determinant of an individual's GH included factors such as the feeling of well‐being, positive sense of self‐efficacy, self‐actualization of intrinsic potentials and capacity of competing in the personal life (Savadpour & Nasiri, 2015). Based on the result of a review and meta‐analysis, the prevalence of mental disorders among Iranian university students based on the general health questionnaire (GHQ) was estimated to be 33% (Zare et al., 2016). Also based on the result of a Swedish study, the prevalence of depressive symptoms among medical students was 12.9% that is significantly higher compared to their peers in the general population (Dahlin et al., 2005).

Compared to childhood, university students exposed to different changes such as physiological, psychological and social changes and struggle with more challenges in the social environment (Rathi & Rastogi, 2007). Although life events considerably changed over time, it is important that MIL be stable in the life (Steger & Kashdan, 2007). In this way, it is stated that there is a significant positive relationship between MIL, involvement in health behaviour in the future and academic achievement (Brassai et al., 2012).

In recent years, MIL converts as interested issues among researchers and is considered as an important component of increased psychological well‐being (Steger et al., 2009). A literature review showed that several studies on MIL were conducted in the Iranian population (Ahmadi et al., 2015; Ahmadi et al., 2016; Dehdari et al., 2013; Khakshoor et al., 2013; Nasiri & Jokar, 2008; Robatmili et al., 2015; Shoshtari et al., 2016). Among the mentioned studies, some of them assessed the relationship between MIL and psychological problems such as depression and anxiety in the students (Ahmadi et al., 2015; Dehdari et al., 2013; Robatmili et al., 2015). Although there is no study that assesses the relation of MIL with students' health status. So this study aimed to investigate the effects of MIL as a predictor of the GH among medical science students.

## 3 METHODS

### 3.1 Design and participants

In this cross‐sectional study, 400 medical sciences students in Mazandaran University of Medical Sciences (MAZUMS), north of Iran, were recruited based on the proportional to size sampling method.

The inclusion criteria were inclination to participate in the study and have no self‐reported psychiatric disorders such as depression and anxiety and no consumption of psychiatric drugs since last year. Cases with failure to complete a maximum of 20% of the questionnaires were excluded from the study.

### 3.2 Data collection and sample size

For data collection, at first, the total number of all the students studying at bachelor of science degree or professional doctorate degree and the total number of students in each major were extracted by assistance of the educational office of the MAZUMS. Then, according to total sample size and by dividing the number of the students in each major (medicine, dentistry, pharmacy, public health, nursing, midwifery and paramedical) per the total number of students in the MAZUMS, the required sample size in each major was calculated. Finally, 400 volunteers' students in the campus of the MAZUMS completed the self‐administrated questionnaires. For all participants, a brief explanation about the objectives of the study and instructions for completing the questionnaires was given. Also, all students were provided written informed consent at the beginning of the study and were assured of the confidentiality of the data.

According to a similar study in Iranian context which stated that approximately 45% of students had favourable GH status (Khazir & Abbasi‐Shavazi, 2019) and by considering confidence coefficient = 0.05, confidence interval = 95%, precision = 0.05 and attrition rate = 5% and by using G Power software, sample size was estimated to 400.

The estimated time for filling out the questionnaires by participants was estimated at 20 min. After collecting the questionnaires, a rapidly checked was performed by researchers to minimize the missing data. Completed questionnaires were placed in separate folders and kept in a locked closet.

### 3.3 Instruments

#### 3.3.1 Sociodemographic characteristics form

This researcher‐made form was included variables such as participants' age, gender, major, grade, marital status, residential place and self‐reported satisfaction of socioeconomic class and their parents' marital status, educational level and job.

#### 3.3.2 Personal meaning profile

To measure the student's perceptions of MIL, the personal meaning profile was administrated (McDonald et al., 2012; Wong, 1998). This questionnaire is a 57‐item tool designed by Professor Paul TP Wong in 1998. It includes seven domains including achievement (16 items), relationship (9 items), religion (9 items), self‐transcendence (8 items), self‐acceptance (6 items), intimacy (5 items) and fair treatment (4 items) on a 7‐point Likert scale (Rathi & Rastogi, 2007). In this tool, each question is scored from 1–7 and it should be noted that there is no category for obtained score in this tool so the final score varies from 57–399 and the higher score indicates the more MIL. The reliability of this scale was confirmed with Cronbach's alpha coefficient of 0.94 (Rathi & Rastogi, 2007). In this study, the reliability of this tool was assessed among twenty university students and the Cronbach's alpha coefficient for each subscale was calculated as follows: achievement = 0.91, relationship = 0.72, religion = 0.77, self‐transcendence = 0.86, self‐acceptance = 0.82, intimacy = 0.77 and fair treatment = 0.73.

#### 3.3.3 General health questionnaire‐28

The GHQ‐28 was designed by Goldberg & Hillier (1979) and considered as a screening tool to identify the probability of psychiatric disorders among the general population (Poorolajal et al., 2017). GHQ‐28 consists of 28 items and four subdomains such as somatic symptoms (items 1–7), anxiety and insomnia (items 8–14), social dysfunction (items 15–21) and severe depression (items 22–28). Each item is accompanied by four‐point Likert scale responses such as “not at all,” “usual,” “more than usual” and “much more than usual,” scoring from 0–3, respectively (Poorolajal et al., 2017).

The GHQ was developed in the United Kingdom in English but it has been translated and used for different researches in the other countries (de Kock et al., 2014). Total scoring of the GHQ28 ranges from 0–84 for the four subscales (lower score showed the better GH status). Various studies assessed the reliability and validity of the GHQ and showed this tool as a reliable and valid measure within various cultural contexts (Goldberg et al., 1997). Based on the results of three Iranian studies which assessed the validity and reliability of GHQ‐28, it showed that the internal consistency of this questionnaire was 0.087–0.91 (Molavi, 2002; Poorolajal et al., 2017; Taghavi, 2001). Also, based on the results of a study regarding psychometric properties of GHQ in college students, the reliability coefficients were calculated in three different methods: test–retest, split half and Cronbach alpha, which were 0.70, 0.93 and 0.90, respectively (Taghavi, 2002). Overall studies regarding the psychometric of this tool in Iran showed the appropriate validity and reliability of this scale among college students (Molavi, 2002; Nazifi et al., 2014; Taghavi, 2002). In an Iranian study, Cronbach's alpha coefficients of the different subscales of GHQ‐28 varied from 0.69–0.88 and its subscales' internal consistency was ranged between 0.28–0.56 (Shayan et al., 2015). Based on the result of a study, the Cronbach's alpha coefficients for subscales of GHQ questionnaire was determined 0.76 for somatic symptoms, 0.84 for anxiety insomnia, 0.61 for social dysfunction and 0.88 for severe depression (Taghavi, 2001).

### 3.4 Statistical analysis

Data analysis was performed by SPSS Statistics 24 (SPSS Inc., IBM Corporation), and to identify missing data, data were checked and cleaned before analysis. To describe quantitative variables such as age descriptive analysis such as mean ± standard deviation (*SD*) and also to describe qualitative variables (such as gender, major, grade, marital status, living in dormitory, parents living together, father educational level, father job, mother educational level, mother job and satisfaction of socioeconomic class), frequency and percentage were applied. To assess the predictors of GH among students, linear regression was used. In linear regression, the relationship between demographic characteristics and MIL as independent variables and GH as dependent was assessed separately. Variables with *p*‐value <.05 in linear regression were entered into the adjusted multivariate linear regression model.

### 3.5 Ethics

This study was approved by the ethics committee of the MAZUMS (Ethical code: IR.MAZUMS.REC = 94.152). All procedures in the study involving human participants were in accordance with the ethical standards of the institutional and/or national research committee and with the Declaration of Helsinki 1964 and its later amendments or comparable ethical standards.

## 4 RESULTS

The mean age of the participants was 21.35 ± 1.94 years and 53% of them were female. Paramedical and medical students were involved in approximately more than half of participants (50.3%) and the majority (55.7%) of the students was in bachelor of science degree. The characteristics of the participants are shown in Table 1.

**Table 1:** Sociodemographic characteristics of the participants (*N* = 400)

Characteristics	Frequency (%)
Age (mean ± *SD*)	21.35 ± 1.94
Gender	
Male	188 (47.0)
Female	212 (53.0)
Major	
Medicine	100 (25.0)
Dentistry	27 (6.8)
Pharmacy	50 (12.5)
Public health	53 (13.3)
Paramedical	101 (25.1)
Nursing	40 (10.0)
Midwifery	29 (7.3)
Grade	
Bachelor of science	223 (55.7)
Professional doctorate	177 (44.3)
Marital status	
Single	323 (81.5)
Married	74 (18.5)
Living in dormitory	
Yes	171 (42.8)
No	229 (57.2)
Parent's living together	
Yes	360 (90.0)
No	40 (10.0)
Father educational level	
Middle school or lower	61 (15.2)
High school	186 (46.5)
Academic	153 (38.3)
Father job	
Employed	196 (49.0)
Self‐employed	204 (51.0)
Mother educational level	
Middle school or lower	112 (28.0)
High school	200 (50.0)
Academic	88 (22.0)
Mother job	
Housewife	247 (61.8)
Employed	153 (38.2)
Satisfaction of socioeconomic class	
Yes	360 (90.0)
No	40 (10.0)

The mean ± *SD* of GH and MIL scores is described in Table 2. The total score of GH was 16.34 ± 6.73. The lower and higher scores were obtained in somatic symptoms (9.73 ± 7.16) and social dysfunction (22.98 ± 11.46) domains, respectively. Also, results showed that 260 (65%) of the students had favourable GH status and other (35%) had one or more disorders in one or more dimensions of the GHQ‐28 questionnaire. In this questionnaire, the total score <24 based on the Likert scoring method is considered favourable GH status. The total score of MIL was 71.50 ± 9.78. From seven domains of this questionnaire, the highest score was reported in the self‐transcendence (78.97 ± 12.39) and the lowest score was in fair treatment domain (65.97 ± 11.75).

**Table 2:** The mean ± *SD* (in percent) of the general health and the meaning of life questionnaires (*N* = 400)

Questionnaires domains	Mean ± *SD*	Minimum	Maximum
General health questionnaire			
Somatic symptoms (Items 1–7)	9.73 ± 7.16	0	43.0
Anxiety‐insomnia (Items 8–14)	12.25 ± 9.84	0	25.0
Social dysfunction (Items 15–21)	22.98 ± 11.46	0	50.0
Severe depression (Items 22–28)	20.40 ± 7.31	0	25.0
Total score	16.34 ± 6.73	0	29.0
The personal meaning profile			
Achievement (16 items)	72.43 ± 10.02	35.0	98.0
Relationship (9 items)	69.65 ± 12.75	25.0	97.0
Religion (9 items)	72.94 ± 10.16	29.0	100.0
Self‐transcendence (8 items)	78.97 ± 12.39	36.0	113.0
Self‐acceptance (6 items)	67.68 ± 10.42	31.0	95.0
Intimacy (5 items)	66.29 ± 15.44	17.0	100.0
Fair treatment (4 items)	65.97 ± 11.75	25.0	100.0
Total score	71.50 ± 9.78	35.0	99.0

As shown in Table 3, factors such as grade, father's educational level, father's job, mother's educational level, self‐reporting of satisfaction about socioeconomic class, and MIL were significantly associated with GH score among medical science students (*p* < .05). These significant factors were entered into the adjusted multivariate linear regression model. In this way, factors such as father's educational level, and the total MIL score were significant predictors of GH score (*R* = .46, adjusted *R* ^2^ = .20, Durbin–Watson = 1.710, *p* < .001). The results showed that students whose father had academic educational level compared to the others had significantly higher GH status (*β* = .159, *p* = .009). Also, the total score of MIL had a positive significant effect on GH status (*β* = .338, *p* < .001). The regression model analysis showed that the total score of MIL was the most important predictors of GH among students in this current study (*R* = .41, adjusted *R* ^2^ = .17, Durbin–Watson = 1.699, *p* < .001).

**Table 3:** Linear regression for predictors of the general health among medical sciences students

Variables (*N* = 400)	Unadjusted univariate linear regression					Adjusted multivariate linear regression model				
	*B*	*SE*	*β*	*t*	*p*‐value	*B*	*SE*	*β*	*t*	*p*‐value
Grade										
Bachelor of science	Ref.	Ref.	Ref.			Ref.	Ref.	Ref.		
Professional doctorate	2.55	0.749	.169	3.41	**.001**	1.27	0.708	.804	1.79	.074
Father educational level										
High school or lower	Ref.	Ref.	Ref.			Ref.	Ref.	Ref.		
Academic	3.73	0.754	.241	4.95	**<.001**	2.46	0.934	.159	2.63	.009
Father job										
Employed	Ref.	Ref.	Ref.			Ref.	Ref.	Ref.		
Self‐employed	−2.50	0.744	−.167	−3.36	**.001**	0.792	0.885	.053	0.894	.372
Mother educational level										
High school or lower	Ref.	Ref.	Ref.			Ref.	Ref.	Ref.		
Academic	3.69	0.892	.203	4.13	**<.001**	0.907	0.899	.050	1.009	.314
Satisfaction of socioeconomic class										
Yes	Ref.	Ref.	Ref.			Ref.	Ref.	Ref.		
No	−6.05	1.22	−.241	−4.96	**<.001**	−1.96	1.22	−.078	−1.60	.109
Total meaning in life	0.081	0.009	.419	9.19	**<.001**	0.065	0.010	.338	6.79	<.001

Significant variables with *p*‐value less than .05 were entered into the adjusted multivariate linear regression model.

## 5 DISCUSSION

The aim of this study was to investigate the predictive role of MIL on the GH among medical sciences students. The health status of students is considered one of the most important public health issues worldwide and an increased level of student's health significantly associated with the high quality of life and subjective life satisfaction (Sreeramareddy et al., 2007).

This study considered four domains of GH including physical symptoms, anxiety and insomnia, social dysfunction and severe depression. Based on the results of the current study, approximately two‐thirds of students had favourable GH status based on the GHQ‐28 questionnaire. The result of a study in Iran also showed that more than 50% of students had a healthy GH status (Hashemian et al., 2015). In this current study, the medical science students with higher father's educational level have better GH status compared to the rest of participants. This result is in line with a study that revealed that the father's educational level was considered as an independent predictive variable for university students' health status in Iranian context (Hashemian et al., 2020). It seems fathers with lower educational levels more likely to force their children for better academic performance, as a result of concerning about their child's welfare in the future, so their students showed psychiatric disorders (Deb et al., 2015). In a study of 1,750, Norwegian undergraduate students indicated that a father's higher educational level was associated with a lower degree of psychological distress (Nerdrum et al., 2006). It can be explained that a higher parent's educational level associated with higher family support and also higher general psychological well‐being. In contrast, in an Iranian study, the GH status of the students was not significantly associated with parents' educational level (Khazir & Abbasi‐Shavazi, 2019).

In the current study, there was a significant relationship between the score of MIL and GH among students. There is growing evidence of a positive relation between MIL and health status in adolescence and youth population (Nielsen & Hansson, 2007). Based on the literature, different studies were conducted regarding different aspects of the health such as psychological, physical and social health (Doğan et al., 2012; Kleftaras & Psarra, 2012). Research has established an association between MIL and the prevalence of psychosomatic symptoms so that an Iranian study investigated the relationship between MIL, depression and anxiety of the medical students showed that there is a significant reverse relationship between the score of anxiety and MIL so that higher MIL associated with lower anxiety scores among students (Dehdari et al., 2013). The literature review revealed that there is a relationship between mental health and academic achievement among university students (Kelvin et al., 1965). The literature review showed that religion/spirituality is an important protective factor against negative health outcomes (Cotton et al., 2006). In fact, religious and spiritual well‐being has an effective protective impact on stress and leads to increased physical and psychological health through having determined valuable goals and MIL (Cotton et al., 2006; Jafari et al., 2010).

Recent studies also revealed the link between MIL with different indicators of physical health and several studies reporting a positive relationship between MIL and self‐reported GH (Brassai et al., 2012; Steger et al., 2015). The results of the study indicated that MIL and significantly associated with life satisfaction and lower psychosocial health problems among adolescents.

Adolescents who have positive expectations for the future are more likely to report high levels of satisfaction in the different life domains (Ho et al., 2010). This shows that if a person perceives his or her life to be meaningful, he or she will feel more psychologically well‐being than those who do not perceive their life to be meaningful (Rathi & Rastogi, 2007). Based on the literature, studies regarding the concepts of the presence of MIL and searching for MIL in students were conducted. The presence of MIL is a protective factor against health risk behaviours (binge drinking, illicit drug and sedative use, unsafe sex and the lack of exercise) and poor psychological health among students (Brassai et al., 2011; Rathi & Rastogi, 2007) and associated with healthy lifestyles (Steger et al., 2015) and life satisfaction, higher self‐esteem and positive affect (Brassai et al., 2011). Studies also have suggested that individuals who are searching for MIL rather than the experience of MIL report poorer physical health and high levels of anxiety (Steger et al., 2006; Steger, Kashdan et al., 2008). A study which evaluates the level of MIL in Japan and the United States revealed that Americans reported greater presence of MIL and Japanese young individuals reported greater search for meaning. In this study, search for meaning was negatively related to presence of meaning and well‐being in the United States and positively related to these variables in Japan and it is concluded that the search for MIL appears to be influenced by culture (Steger, Kawabata et al., 2008).

### 5.1 Limitations and strengths

The present studies had some limitations and strengthen. This study is one of the limited published studies about the MIL and its role on GH status of medical science students. The limitation of this study was the cross‐sectional design of the study and the self‐report methods to assess the student's GH so the depression domain of GHQ‐28 may not be reliable to evaluate the mental status of students. Also, generalization of the results from this sample to the general population of Iranian university students is limited due to conducting this project in medical sciences students.

## 6 CONCLUSION AND IMPLICATION FOR CLINICAL PRACTICE

Assessment of the GH is an important issue among medical sciences students since these students are responsible for maintaining and promoting the health status of the community in the future. Due to the lack of research about association of MIL and GH among university student in Iran, the findings of the current study make a new conceptual foundation regarding the role of MIL as an important predictor of the GH in this age group. As the importance of higher MIL and GH for meaningful and successful life in youth population, designing and implementing high‐quality studies regarding the evaluation of students' GH and also considering appropriate interventional studies regarding the role of MIL on students' health are important and effective action.

## CONFLICT OF INTEREST

The authors declare that they have no conflict of interest.

## AUTHOR CONTRIBUTIONS

ZSH designed the study and analysed the data; MA collected the data and contributed to the study design and wrote the paper. ZHG and RM contributed to the study design. All authors made a substantial contribution to the writing of the paper draft and met the four criteria for authorship recommended by the International Committee of Medical Journal Editors.

## Data Availability

The data of this study are available upon the journal request.
